# Role of mucosal IgA antibodies as novel therapies to enhance mucosal barriers

**DOI:** 10.1007/s00281-024-01027-4

**Published:** 2024-11-20

**Authors:** Peng Gao, Naoki Morita, Reiko Shinkura

**Affiliations:** Laboratory of Immunology and Infection Control, Institute for Quantitative Biosciences, The University of Tokyo, 1-1-1 Yayoi, Bunkyo-Ku, Tokyo, 113-0032 Japan

**Keywords:** Immunoglobulin A, Vaccines, Allergy, Gut microbiota, Mucosal immunity

## Abstract

To prevent infection, the experience of the recent severe acute respiratory syndrome coronavirus 2 (SARS-CoV2) pandemic has led to recognition of the importance of not only vaccines but also the strengthening of mucosal barriers by secretory immunoglobulin A (IgA). Strong mucosal barrier provided by IgA is also possible to prevent allergies and chronic inflammatory conditions in the intestinal tract, since it can protect foreign enemies or antigens at the first line of defense before their invasion. Therefore, it is important to understand the role of IgA antibodies secreted by the mucosa of the body. In this section, we discuss the role of mucosal IgA antibodies in relation to three disease states: control of intestinal microbiota, protection against infection, and allergy. In addition, we provide the evidence in which the quality as well as the quantity of IgA is critical for disease prevention. Therefore, we discuss about novel strategies to enhance mucosal barriers by induction of high-quality IgA.

## Introduction

Vaccines are effective against specific infectious diseases such as the severe acute respiratory syndrome coronavirus 2 (SARS-CoV2), polio, and measles, but what is more important is to develop the ability to protect our body against a wide variety of pathogens in the steady state. To do so, having a healthy mucosal barrier is the most important factor in preventing disease, because most pathogens invade from the mucosal surfaces [1]. Standard current vaccines are injected intramuscularly, which activates the systemic immune system but not the mucosal immune system. Therefore, it is necessary to consider how to strengthen the mucosal immune barrier from the aspect of healthy longevity and countermeasures against new infectious pandemics in the future. We should also consider not only vaccine fortification but also steady-state mucosal barrier fortification.

In this aspect, the intestines are important organs, since they are exposed to foreign enemies over a large area. In the intestine, as the first line of host defense, many antibodies and immune cells work to prevent invasion of foreign enemies [2, 3]. In this chapter, immunoglobulin A (IgA) antibodies, which play important roles in mucosal defense, are reviewed in relation to normal microbiota maintenance, protection against infection, and allergic reactions.

### Mucosal IgA and commensal microbiota

The well-known antibodies are IgG antibodies, which are the most abundant in blood, and almost all the antibodies developed as therapeutic antibodies are IgG antibodies. IgG antibodies are Y-shaped proteins consisting of two heavy and two light chains. The two arms of the Y are called the variable regions, and they are different sites for different types of antibodies. They recognize and bind antigens at their tips (antigen-binding sites). The region corresponding to the Y-shaped foot, called the constant region, has the same structure for each isotype (IgM, IgG, IgE, and IgA) and functions by binding to receptors specific for that isotype.

The intestinal mucosa secretes 3–5 g of IgA antibody per day into the gut lumen in adults, making it the most abundant antibody produced in the body. It is reasonable that such large amounts of antibody must be released into the intestinal lumen daily to protect the entire intestinal mucosa from invasion by foreign bodies [4, 5]. Unlike IgG antibodies, IgA antibodies secreted by the intestinal and other mucous membranes are dimers of two Y-shaped chains linked by a short peptide called the J chain [4]. In addition, when secreted from the mucosa, they take the form of secretory IgA antibodies with secretory component wrapped around them [4, 5]. These secretory IgA antibodies are present not only in the intestinal tract but also in the mucosal surfaces of the whole body, including the oral cavity, nasopharynx, respiratory tract, lacrimal gland, mammary gland, and genitourinary tract, and are an important component of mucosal defense against various foreign substances [4, 5].

IgA antibodies that act in the mucosa, such as intestinal IgA antibodies, have a wide range of antigen specificities, and it is known that a single type of antibody can respond to multiple antigens, in contrast to IgG antibodies that specifically recognize almost a single antigen [6–9].

However, while recognizing multiple antigens, intestinal IgA antibodies, for example, distinguish intestinal bacteria and do not exclude the beneficial bacteria such as *Lactobacillus casei*, which are commonly used as probiotics [6]. It has also been reported that IgA antibodies assist for some beneficial bacteria to colonize the mucus layer on the intestinal mucosal surface [10]. On the other hand, among the commensal microbiota, IgA antibodies bind to enteritis-induced bacteria such as *Escherichia coli* and control the occurrence of enteritis by quiet elimination without inflammation [11]. In this way, IgA antibodies are thought to play an important role to maintain the healthy gut environment. However, IgA antibodies can trigger an inflammatory response to eliminate an enemy when necessary against invasion of a pathogen including viruses [11]. The way in which such a clever immunity can discriminate against an enemy is still not well understood, but it is a very interesting immunological question.

The number of patients with IgA antibody deficiency is the highest among congenital immune deficiency diseases, and is said to be about one in 500 in Europe and the United States.　A recent large cohort study reported a particularly high prevalence of autoimmune diseases and inflammatory bowel diseases (IBD) (ulcerative colitis and Crohn's disease) in IgA deficiency [12]. The increased prevalence of these diseases may have been due to dysbiosis (imbalanced gut microbiota), which may have resulted from a lack of intestinal IgA antibodies and a consequent inability to control intestinal microbiota [13, 14]. Recent studies discussed the implications of IgA deficiency on intestinal immunity, especially highlighting the connection between the lack of IgA and altered intestinal microbiota (dysbiosis), leading to the gut inflammation [13, 14]. These results indicate that the amount of IgA antibody is important for the control of intestinal microbiota.

In addition, the quality, in other words the affinity of IgA antibody, should be considered for the gut homeostasis [15]. What we need to clarify here is that high affinity does not necessarily require many mutations. To produce high-affinity IgA, B cells must undergo germinal center (GC) reactions to accumulate affinity. However, more mutations do not always correlate with higher affinity, as somatic hypermutation (SHM) occurs randomly and too many mutations may disrupt the antibody structure itself. The previous reports have demonstrated that as aging dysbiosis is seen. Sugahara et al. have shown that the relative abundance of Bifidobacteriaceae decreased and that of Enterobacteriaceae increased in the elderly (average age: 76 years, advanced aging) compared with the adult (average age: 35 years), despite normal fecal IgA levels in both the adult and the elderly [16]. By IgA-sequencing analysis, it is revealed that adult IgA antibodies bound strongly to Enterobacteriaceae, whereas elderly IgA antibodies did not, indicating that low binding ability of IgA antibodies from the elderly to Enterobacteriaceae allows them vulnerable to growth in the intestinal lumen [16]. A decrease in the number of Bifidobacteriaceae and an increase in the number of Enterobacteriaceae have been reported in both human and mouse IgA deficiency and in patients with IBD [17–19]. These results strongly suggest that not only a decrease in the quantity of IgA antibodies but also a decrease in their quality may be responsible for the development of dysbiosis.

Another example is a study in which fecal IgA antibodies from patients with IBD were tested for their ability to bind to intestinal bacteria [20]. When IgA-sequencing analysis was performed in a manner like the study of IgA antibodies in the elderly [16], intestinal IgA antibodies produced by IBD patients bound strongly to bacteria, especially Lactobacillaceae, that were different from those of healthy individuals. This suggests that altered IgA quality in patients with IBD may be one of causes of dysbiosis, although the underlying mechanism is unknown.

From the other side, we have shown that high-affinity IgA antibodies are required for the regulation of commensal gut microbiota. AIDG23S knock-in mice were generated by introducing a mutation into a molecule (activation-induced cytidine deaminase, AID) essential for mutation of the antibody gene [15]. These mice have enough IgA antibodies in the intestinal tract but can produce only low-affinity IgA antibodies because no mutation occurs [15]. The mice had enlarged Peyer's patches, the mesenteric lymph nodes, and increased GC B cells [15]. When the commensal microbiota was reduced by administration of antibiotic cocktail, this over-immune response was improved, suggesting an over-immune response due to stimulation from the commensal microbiota in AIDG23S mice. In fact, in AIDG23S mice, the composition of intestinal microbiota was altered and dysbiosis was observed [21]. Thus, the presence of enough amounts of IgA antibodies in the intestinal lumen is not enough for normal microbiota control, whereas high-affinity IgA antibodies are required for normal microbiota maintenance.

In addition to the microbiota control, the importance of antibody somatic mutations has been clearly demonstrated when cholera toxin, a toxin derived from *Vibrio cholerae*, is administered orally to AIDG23S mice [15]. AIDG23S mice were more susceptible to a small dose (60 µg) of cholera toxin than were wild-type mice. Although wild-type mice had never been exposed to cholera toxin, some of the high-affinity IgA antibodies against other bacteria cross-reacted with cholera toxin, suggesting that they provided better protection against cholera toxin than AIDG23S mice [15]. These results indicated that the IgA antibodies necessary for intestinal bacterial control are those that react with a wide variety of bacteria and have high affinity (strong binding), which may protect the host from unpredictable invading pathogens.

From these observations, two strategies are considered to maintain the intestinal microbiota in healthy condition. One is to raise the affinity of intestinal IgA antibodies by educating the immune system through vaccination, or continuous commensal stimulation to enhance germinal center mutation process to produce high-affinity and poly-reactive antibodies. The second is to replenish the intestinal tract by drinking high-affinity IgA antibodies that react with a wide variety of bacteria. We think of high-affinity IgA antibodies as a potential treatment for dysbiosis in patients with the inability to produce high-affinity IgA antibodies, in a similar situation of AIDG23S mice [15] and IBD patients as described above [20]. In particular, as recent reports have mentioned, dysbiosis has been found not only to cause enteritis, but also to cause metabolic, neurologic, and circulatory diseases in organs that do not directly associated with intestinal bacteria [22–26]. How to intervene in the intestinal microbiota and treat dysbiosis is an important issue that will lead to preventive treatment in the next generation, and the importance of IgA antibodies in the intestinal tract will increase more and more.

### Mucosal IgA and defense against infection

With the recent COVID19 pandemic, the importance of not only IgG antibodies in blood but also mucosal IgA antibodies that can protect the host against the invasion of pathogens has been widely recognized [27–30]. As described above, mucosal secreted IgA antibodies in the respiratory tract and the intestinal tract are multimers. Therefore, to prevent contact between antigens and host cells, they have a greater antigen-binding force than IgG antibodies [31], and because of their molecular size, they are effective in preventing infection by binding to cover up the nearby antigenic variation that occur in the antigen molecules of viruses [32]. This property is even more effective with IgM antibodies, which constitute even larger molecules than IgA antibodies, and the development of mucosal administration of IgM antibodies has also been proposed as a therapeutic antibody [32, 33].

In addition to their physical size, mucosal IgA antibodies are also known to have a wider range of antigen specificities than IgG antibodies, and this is illustrated by the cross-reactivity of the antibodies that provides protection against the cholera toxin as described above [6, 9]. It is of great interest to know by what criteria B cells have been selected that produce antibodies capable of such polyreactive antigen recognition at the mucosal site.

As noted above, the studies in AIDG23S mice have shown that high-affinity, poly-reactive intestinal IgA antibodies in wild-type mice are made possible by the accumulation of mutations in antibody genes [15]. Indeed, when the frequency of mutations in intestinal B cells of wild-type mice was examined, it increased with increasing age (but before advanced aging. Comparison in somatic mutation frequency was done between 8- and 20-weeks old mice. (Unpublished data)), suggesting that intestinal B cells are continually educated by ceaseless stimulation from the gut bacteria. In the gut, those accumulation of mutations followed by selection of B cells occur in GC of Peyer's patches, mesenteric lymph nodes, and cecum [34–37]. A single clone of B cells is known to undergo repeated GC responses, a process that may link accumulation of mutations and selection of high-affinity antibodies, leading to an enhanced mucosal immune barrier [34–37]. Therefore, to enhance the mucosal IgA response, one solution is to find a way for intestinal B cells to efficiently migrate to germinal centers. In this way, mucosal vaccines that enhance mucosal IgA antibodies are possible, although an effective mucosal vaccination has not yet been commercialized.

The principle to evaluate a vaccine is based on whether it can induce high-affinity antibodies and immune memory. Mucosal poly-reactive IgA antibodies may have a strong effect to neutralize an even unexpected pathogen in the future, as described above in cholera toxin challenge [15]. However, the vaccines for IgA induction in the mucosal immune system have been slow to make significant progress due to the lack of a safe and reliable delivery method. For example, traditional oral delivery methods often result in vaccines being digested by gastric fluids, which leads to the suboptimal stimulation of B cells in Peyer’s patches, the induction site for IgA in intestinal immune system [38]. However, increasing evidence suggests that mucosal vaccines targeting microfold (M) cells can effectively transport antigens to mucosa-associated lymphoid tissues, thereby eliciting specific immune responses against the antigen [39, 40]. Thus, developing safe and effective adjuvants for activating B cell responses in the intestinal tract is currently in progress with effort.

Oral and nasal routes are common methods for mucosal immunization [41, 42]. Compared to traditional systemic immunization, oral immunization typically requires longer response times and more immunization sessions. Lycke's group demonstrates that using cholera toxin as an adjuvant for oral immunization, with at least three immunizations, can successfully induce high-affinity antigen-specific gut IgA responses [43]. When we think about safety using lipopolysaccharide (LPS), a typical endotoxin, only 1 to 4 ng/kg body weight for intravenous administration is the maximum safe dose, but even 300 mg/kg is an acceptable amount for oral administration in mice without significant side effects [44–48]. At such high doses for oral administration, mouse body weight remained unchanged, and there was no increase in inflammatory cytokine level in serum. It suggests that using adjuvants in oral immunization is indeed a very safe and effective method to activate the mucosal immune system in the gut.

Intranasal immunization is another promising convenient approach for vaccination based on mucosal immune system [49–51]. It can activate mucosal immune responses to generate the high-affinity IgA at the respiratory tract, which is vital in defending against the specific respiratory infections and pathogen invasions, like SARS-CoV2 [49–51]. However, there are concerns about potential safety risks of intranasal immunization, since antigens and adjuvants may be inhaled through the nasal cavity and reach the brain. Therefore, achieving safe and efficient activation of mucosal immunity require further deep-research and investment.

**Fig. 1:**
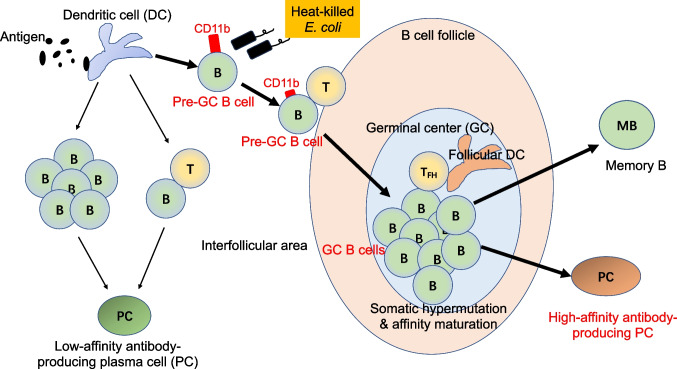
Heat-killed *E. coli* stimulates B cells to express CD11b transiently and enter GCs to produce high-affinity antibodies. CD11b is a newly identified pre-GC B cell surface marker. Heat-killed bacteria such as *E. coli* or *Salmonella*, but not *Bifidobacterium breve*, induce CD11b on the activated B cells and help them to migrate into GCs. Thus, heat-killed *E. coli* can be utilized as an effective mucosal adjuvant.

Based on the above insights, we are committed to developing an efficient and safe mucosal adjuvant from the perspective of efficiently directing B cells to GC. Through our research on pre-GC B cells that is ready to enter the GC in Peyer’s patches, we have demonstrated that some Toll-like receptor (TLR) ligands, particularly TLR2 and TLR4 ligands, can effectively stimulate B cell proliferation and activation to let them enter the GCs [52]. Oral administration of TLR2/4 ligands can significantly increase the number of GC B cells in Peyer’s patches within two days [52], which is necessary for production of high-affinity IgA antibodies. Beyond traditional Pam3CSK4 (TLR2 ligand) and LPS (TLR4 ligand), we have attempted to use heat-killed *E. coli* as an adjuvant for oral immunization, since heat-killed *E. coli* contains various components capable of simultaneously activating both the TLR2 and TLR4 on B cells (Fig. 1) [52]. On the other hand, since an *E. coli* strain that we selected is one of commensal bacteria in the human gut, we believe that oral administration of heat-killed *E. coli* should result in fewer side effects and a safer induction of high-affinity intestinal IgA. Through oral immunization with spike protein (SARS-CoV2) as an antigen and heat-killed *E. coli* as an adjuvant, we could induce fecal IgA antibodies not only with high-affinity against spike protein but also validated high-neutralization ability which has been shown through pseudo-virus infection assays (unpublished data). This is an example; however, the development of efficient mucosal vaccines is an important challenge that will lead to the prevention of future pandemics caused by novel pathogens.

### IgA and allergic diseases

Here we describe the contribution of IgA in allergic disease development, including the induction of the pathogenesis of allergic disorder as well as the immune tolerance to the allergen at the early life stage. We also discuss the therapeutic approach of allergen immunotherapy, which induces allergen-specific IgA production.

Allergic diseases are one of the immune-mediated common chronic inflammatory diseases, caused by persistent or repetitive exposure to allergens at the mucosal site. In recent years, allergic patients have been increasing globally and show much more severe symptoms. Allergic disorder, including asthma, rhinitis, food allergy, and hay fever, is associated with increasing environmental allergen-specific IgE in the serum [53]. Allergens can penetrate the mucosal tissues through the damaged epithelium and be captured by myeloid cells in the mucosal sites for a sampling of luminal antigens. Some allergens have the function of proteases, which directly induce epithelial disruption through the shedding of epithelial cell tight junction. Antigen-captured myeloid cells are activated and migrate to the mucosal-associated lymphoid tissues. Myeloid cells present allergen-derived peptides in the context of major histocompatibility complex (MHC) class II molecules to naïve T cells in the lymph nodes. Antigen-presented naïve T cells develop into T helper 2 (Th2) cells by signaling of interleukin 4 (IL-4), which is mainly produced by a broad range of cells, including, basophils, mast cells, eosinophils, dendritic cells, and macrophages [53]. Th2 cells contribute to the production of IL-4, IL-5, and IL-13 at the mucosal site to amplify the allergic responses.

Recently, type 2 innate lymphoid cells (ILC2) have also been identified as one of the major producers of IL-4 and IL-13 in the development of allergic diseases. Damaged-epithelial cells-derived IL-25 and IL-33 rapidly induce secretion of IL-4 and IL-13 in ILC2 cells, indicating that ILC2 activation is induced in the early phase of allergy [54]. IL-4 and IL-13 derived from Th2 and ILC2 cells induce immunoglobulin class-switch recombination from IgM to IgE in B cells. IgE also acts on the fast defense molecules like another antibody isotype against invading pathogens such as nematodes [55]. The hallmark of the allergic response, exposure to exogenous allergens makes a complex with IgE which binds on the cell surface of mast cells and eosinophils through the Fc epsilon receptor (FcεR). Once the antigen binds to IgE and crosslinks FcεR, FcεR signaling induces activation and degranulation of mast cells and eosinophils to release immediate inflammatory mediators such as the histamine, leukotrienes, prostaglandins, protease, and pro-inflammatory cytokines. This inflammatory response leads to symptoms in different target mucosal tissues associated with allergic responses, the skin (atopic dermatitis), the nose (rhinitis, hay fever, and sinusitis), the lung (asthma and eosinophilic pneumonia), the gut (food allergy and celiac disease) after few minutes later [53]. IgE-expressing B cells and IgE producing plasma cells exist less than 1% among all B cells and plasma cells, respectively, in mucosal tissues including upper respiratory tissue, lower respiratory tissue, and intestine in healthy subjects. In contrast, patients with allergic disorders preserve approximately 4% of IgE-expressing B cells among all B cells and more than 10% of plasma cells among all plasma cells in the mucosa [56]. However, it remains unclear how allergic responses selectively develop Th2 cells which induce IgE production from B cells and plasma cells. In addition, although IgA are well known to function at the mucosa, the contribution of IgA to allergic diseases is not well understood.

Selective IgA deficiency is one of the common human primary immunoglobulin defects associated with the lack of IgA in the serum (less than 7 mg/dL of IgA but normal serum level of other immunoglobulins in subjects over 4 years of age) [57, 58]. Several genetic defects have been proposed for selective IgA deficiency such as disorder in B cell maturation, T cell development or production of cytokines. Emanuela et al. reported that selective IgA deficiency patients have a missense mutation in one allele of *TNFRSF13B* (encoding transmembrane activator and calcium-modulator and cytophilin ligand interactor (TACI)), which is the receptor for tumor necrosis factor ligand superfamily, member 13b (encoding B cell activating factor belonging to the tumor necrosis factor (BAFF)) and a proliferation-inducing ligand (APRIL) [59]. Since signaling of BAFF and APRIL through TACI in naïve B cells contributes to IgA class-switching, a mutation in *TNFRSF13B* can result in selective IgA antibody deficiency. According to the human subjects, APRIL-deficient mice showed impaired levels of IgA production in the serum and intestine [60].

It is well studied that selective IgA deficiency patients are associated with allergic diseases and various atopic symptoms in addition to autoimmune diseases, as described above [61]. Yazdani et al. estimated that more than 40% of the first manifestation in IgA deficiency patients is an allergic disorder [61]. Therefore, the manifestation of allergic disorders is also an important suspicion in as same as patients with recurrent infections for diagnosis of IgA deficiency. A recent larger epidemiological analysis performed by Moschese et al. identified that 18.45% of selective IgA deficiency patients have allergic rhinitis, 12.6% have atopic dermatitis, and 10.67% have allergic asthma [62]. Other epidemiology studies of selective IgA deficiency also demonstrated that IgA plays a pivotal role in the prevention of allergic disease [63, 64].

Although selective IgA deficiency patients tend to develop allergic diseases, mechanisms of the onset of allergic disorders remain in argument. Selective IgA deficiency patients show high blood levels of IL-4 and IL-13 [65], indicating the dominant Th2 response, cytokine imbalance of unknown cause. One possibility is that the absence of mucosal IgA weakens the mucosal barrier and allows entry of allergens through the mucosa, resulting in an increase in circulating allergens and activation of immunity. In addition, IL-4 and IL-13 are known to enhance the epithelial permeability of antigens by regulating the expression of tight junction-related molecules in epithelial cells [66, 67]. It allows us to speculate that high levels of IL-4 and IL-13 in IgA deficiency provide positive circularization in the pathogenesis of allergic disorders through increasing antigen permeabilization. However, it has not been properly examined whether mucosal allergens are captured by luminal-secreted allergen-specific IgA, therefore it needs to be studied in the future.

On the other hand, it is well known that IgA deficiency induces dysregulation of commensal bacteria in the gut dysbiosis as described above [13, 14, 17]. Another possibility is that gut dysbiosis may affect the IgE production and pathogenesis of allergic diseases. There are some reports about the relationship between commensal bacteria and IgE production in the mouse study. Cahenzli J et al. described that germ-free mice showed high serum levels of IgE [68]. IgE class-switched B cells were increased in neonatal germ-free mice in CD4^+^ T cell- and IL-4-dependent manner. Therefore, oral-induced systemic anaphylaxis is also exaggerated in germ-free mice compared with SPF animals, which is associated with elevated serum IgE levels and increasing surface-bound IgE on mast cells in the gut. This report indicated that the commensal bacteria in the gut is necessary for the maintenance of healthy IgE levels. In another article, Amano S et al [69]. reported that MyD88-deficient mice enhance systemic IgE production. In MyD88-deficient mice, not only serum IgE levels, but also IgE-producing plasma cells in the spleen, mediastinal lymph nodes, and mesenteric lymph nodes are increased compared to wild-type animals [69]. Although *Lactococcus formosensis* mainly colonizes in the lung mucosa of wild-type mice, *Streptococcus azizii and Streptococcus danieliae* dominated in the lung bacterium in MyD88-deficient mice. Intratracheal administration of *S. azizii* enhanced IgE production in the lung mucosa in a dendritic cell-dependent manner. This result indicates that not only intestinal commensal bacteria but also lung-colonizing bacteria affect the production of IgE in the serum and mucosa. These data suggest that dysbiosis in the mucosa mediated by IgA deficiency induces high levels of IgE antibody production.

It has been reported that maternal milk-derived IgA antibody plays an important role in the prevention of allergic diseases in the early life of offspring [70]. The first milk has the highest level of IgA compared with mature milk during lactation. Several studies described that IgA in maternal milk recognizes airborne, pollen, and food allergens [70, 71]. Orivuori et al. analyzed whether total IgA levels or transforming growth factor-β1 (TGF-β1) in breast milk were inversely associated with an atopic dermatitis development in the early life stage of human offspring. They collected 610 milk samples and analyzed the concentration of IgA and TGF-β1. Atopic status was diagnosed by allergen-specific IgE concentration in the blood samples at ages 4 and 6 years. IgA antibodies but not TGF-β1 in breast milk were inversely associated with the development of atopic dermatitis at the ages until 6 years [72]. In the mouse study, maternal milk-derived IgA plays a pivotal role in the induction of RORγ^+^ regulatory T cells (Tregs) in the intestine of offspring. In particular, there is a strong correlation between the parentage of maternal IgA-binding commensal bacteria and the number of RORγ^+^ Tregs in the intestine. This result indicates that maternal IgA may prevent the onset and/or exacerbation of food allergy in offspring through induction of RORγ^+^ Tregs [73]. However, it remains unclear how maternal milk-derived IgA-binding commensal bacteria induce RORγ^+^ Tregs of the intestine in offspring.

More recently, therapies have been developed to suppress IgE production by regulating immunity. Allergen immunotherapy is the repeated administration of allergen or allergen-related products to patients with allergic disorders through sublingual and subcutaneous routes to induce long-term immune tolerance which is associated with decreasing allergen-specific IgE production [74]. For hay fever patients, allergen immunotherapy treatments transiently enhanced pollen-specific IgE production. Over several years of treatments, the production of allergen-specific IgE gently declines and symptoms of hay fever are gradually attenuated.

In addition to pollen-specific IgE, allergen-specific IgA1 and IgA2 are produced by subcutaneous and sublingual allergen immunotherapy [75]. Both administration routes successfully induced allergen-specific IgA1 and IgA2 in the serum after 2–6 months later. These IgA1 and IgA2 are increased in the blood of subcutaneous and sublingual allergen immunotherapy-treated patients compared with an untreated group. Pilette C et al. analyze that cross-linking between allergens and allergens-specific IgA2 in the cell surface of immune cells through the Fc α receptor I (FcRαI) which mice lack, affects the production of cytokines. Cross-linking of IgA2 with allergens through FcRαI in the cell surface of monocytes enhanced the production of the IL-10, indicating that subcutaneous allergen immunotherapy may induce protective IgA production and tolerance to allergens [76]. Although allergen immunotherapy induced allergen-specific IgA1 and IgA2 in the serum of patients, the correlation between clinical outcome and allergen-specific IgA production in the serum is insignificant. We supposed that allergen immunotherapy mainly produces allergen-specific IgA in the blood but not the mucosal site. To protect against penetration of allergens, the induction of allergen-specific IgA in the mucosa is necessary for the further strategy for allergic disorders.

**Fig. 2:**
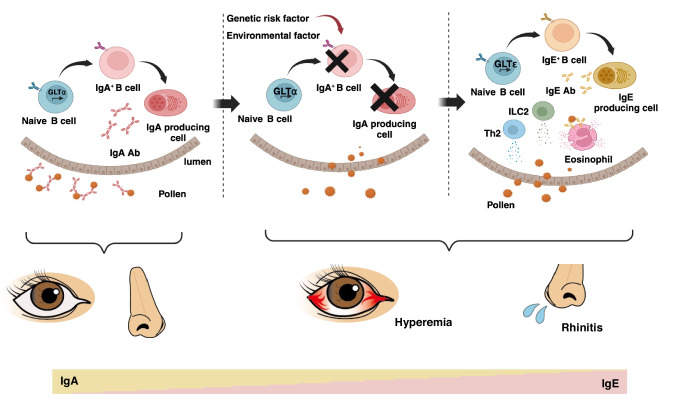
IgA plays a barrier in preventing the invasion of allergens in the mucosa. Although IgA deficiency is highly associated with allergic diseases in not only the intestines but also other mucosal tissues, such as rhinitis and hyperemia, the pathogenesis of these diseases in patients with IgA deficiency remains unclear. We hypothesize that allergen-specific IgA prevents the penetration of allergens into our body to initiate allergic responses. Therefore, IgA-deficiency or reduction of allergen-specific IgA shows amplified allergic responses, including. production of IgE, Th2-related cytokines, and infiltration of eosinophils and mast cells.

It is well known that IgE is very important for the pathogenesis of allergic disorders. In contrast, the functional role of IgA in allergic diseases is poorly understood. The clinical and basic studies suggest that allergen-specific IgA could interrupt the penetration of epithelial cells in the lumen and binding between allergens and allergen-specific IgE. However, we have limited knowledge of whether induction of allergen-specific IgA is helpful for the treatment of allergic diseases. Finally, it may lead to a novel therapeutic approach that induces allergen-specific IgA production to prevent penetration of allergens, especially in the mucosal site (Fig. 2).

## Conclusions

**Fig. 3:**
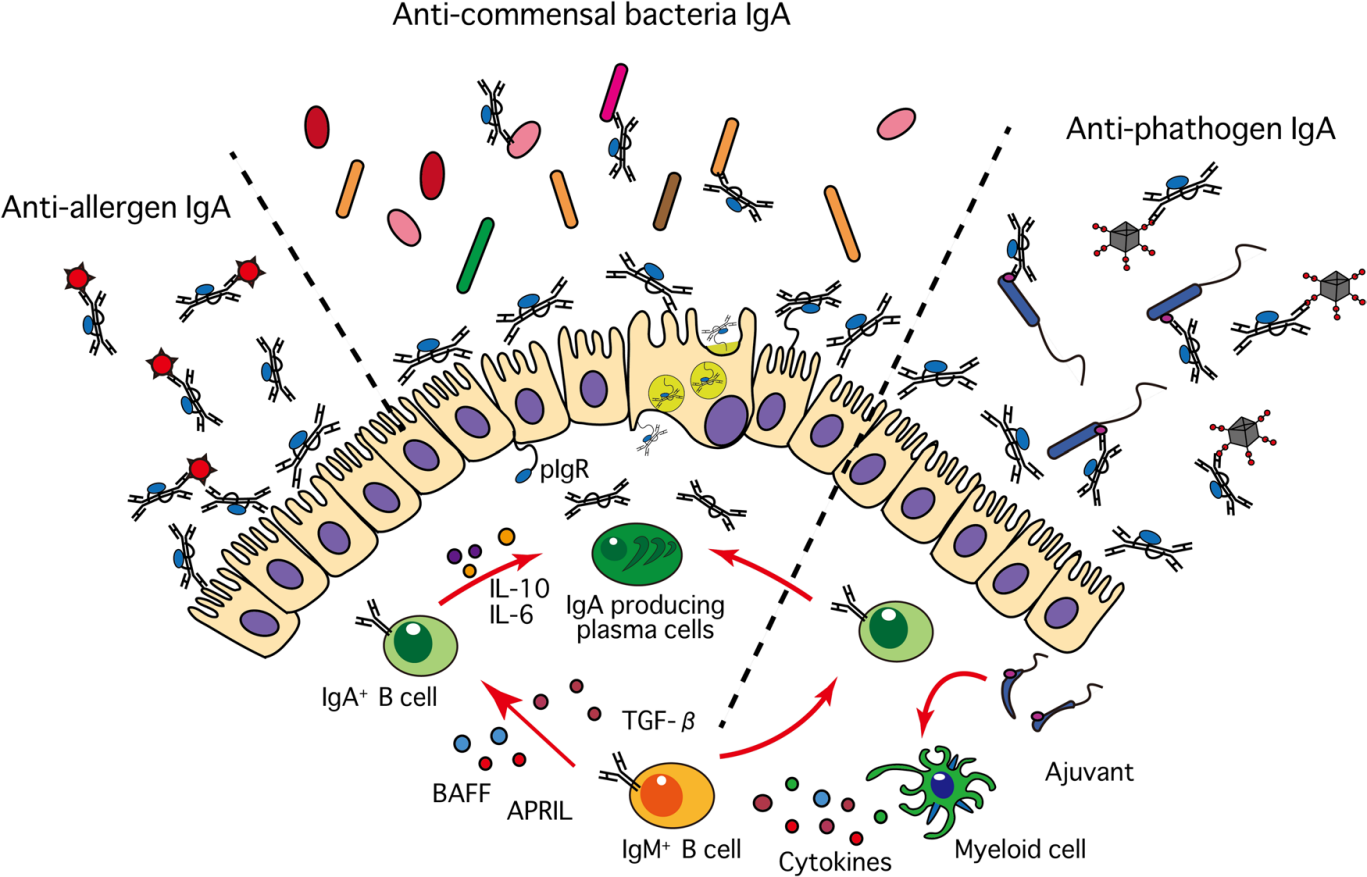
IgA as a guardian deity of the mucous membranes. Working at the mucosa, IgA, together with a variety of secreted antimicrobial molecules, is important in maintaining mucosal barrier function and is highly selective in distinguishing friend from foe. Among non-self substances such as bacteria, fungi, viruses, pollen, and food, it identifies undesirable partners for the host, prevents invasion through the mucosal surface, and contributes greatly to the maintenance of homeostasis and health of the host.

In this section, we have looked at the role of mucosal IgA antibodies in various disease states (Fig. 3). What individual IgA clones recognize and what effects they have on pathogens remains to be studied. The development of IgG antibody precedes as a molecular target medicine, and it occupies the important position in the medical treatment. It is expected that the antibody treatment which utilized the distinct characteristics of the IgA antibody will be used practically in future.

In fact, previously, we have shown that a single IgA clone is polyreactive to intestinal bacteria, which recognizes and inhibits the growth of pathogenic bacteria in vitro, while having no effect on *Lactobacillus casei* used as probiotics [6]. The molecular mechanism by which this IgA clone inhibits growth of individual bacteria still needs further investigation. However, oral administration of this monoclonal IgA antibody to mice has been shown to alter the intestinal microbiota and improve conditions such as enteritis and colonic polyposis in mouse models [6, 77, 78].

In this way, IgA antibodies do not only target a single pathogen, but also have a function to regulate the whole intestinal microbiota. Thus, IgA antibodies are important to intervene in the disease state of dysbiosis that cannot be clearly defined. This could lead to a higher level of protection against infectious diseases and, together with vaccines, could be an effective means of countering future pandemics. In this section, although we mainly focus on IgA function for microbiota, the role of IgA in intestinal fungi and helminth has been discussed [79–82]. Research on mucosal IgA should be advanced to maintain the symbiotic relationship between microorganisms (including viruses) and hosts, and to realize healthy longevity.

## Data Availability

N/A.
